# Acute caecal volvulus during a 90 km ultramarathon: A case report

**DOI:** 10.17159/2078-516X/2026/v38i1a27542

**Published:** 2026-10-15

**Authors:** SG Feris, MR Lukusa, G Pauselli

**Affiliations:** 1 School of Clinical Medicine, College of Health Sciences, University of KwaZulu-Natal, Durban, South Africa; 2 Section of Exercise Science and Sports Medicine, Faculty of Health Sciences, University of the Witwatersrand, Johannesburg, South Africa

**Keywords:** endurance running, abdominal pain, bowel obstruction, mobile caecum, hemicolectomy

## Abstract

**Background:**

Caecal volvulus is an uncommon cause of large bowel obstruction that occurs in patients with a congenitally mobile caecum. It is rarely reported in association with endurance exercise.

**Objectives:**

To describe an acute caecal volvulus precipitated during a 90 km ultramarathon and to highlight the diagnostic and management considerations relevant to sports and exercise medicine.

**Case:**

A 51-year-old female runner developed acute severe right-sided abdominal pain and vomiting while running the Comrades Marathon. Contrasted computed tomography demonstrated a markedly distended caecum consistent with caecal volvulus. She underwent emergency laparotomy, right hemicolectomy and ileocolic anastomosis. A transient rise in troponin with a structurally normal echocardiogram was attributed to demand ischaemia. She recovered uneventfully.

**Conclusion:**

Caecal volvulus should be considered in an endurance athlete presenting with acute right-sided abdominal pain. Early cross-sectional imaging and prompt surgery are key to a good outcome.

Volvulus of the colon is the third most common cause of large bowel obstruction worldwide, of which caecal volvulus is the less common form, typically affecting younger women with a congenitally mobile caecum that has incomplete peritoneal fixation.^[1]^ Although gastrointestinal symptoms are among the most frequent complaints in endurance runners, frank surgical emergencies during a race are rare.^[2]^ Caecal volvulus precipitated by long-distance running has only occasionally been described.^[3]^ We report an acute caecal volvulus that developed during a 90 km ultramarathon, requiring emergency resection.

## Case presentation

A 51-year-old female recreational distance runner with no chronic illness and no previous abdominal surgery developed severe, colicky right-sided abdominal pain at approximately 75 km into the Comrades Marathon, a 90 km ultramarathon in KwaZulu-Natal, South Africa. She completed the up-run (from Durban to Pietermaritzburg) despite the pain, finishing in 9 hours 52 minutes. With unremitting pain, inability to stand and the onset of vomiting, her partner presented her directly to the emergency centre for further evaluation, bypassing the finish line emergency tent. On arrival, she was in severe pain, haemodynamically stable and afebrile (heart rate 84 beats per minute, blood pressure 175/68 mmHg, respiratory rate 20 breaths per minute, oxygen saturation 98% on room air). She was triaged as urgent (orange) using the South African Triage Scale (SATS), requiring evaluation by a healthcare provider within 10 minutes of arrival.

On history, obtained from the patient, she had run multiple Comrades Marathon events. This was her index presentation to an emergency centre due to an endurance race adverse event, and she had never experienced pain of this nature before. She had no significant past surgical or medical history. Her nutrition plan consisted of a mixture of carbohydrate drinks, gels and roadside solids – as was typical for her and unchanged from past events. She reportedly experienced GI symptoms early in the race (abdominal cramps and diarrhoea), following which she self-medicated with >5 Loperamide tablets (a u-opioid receptor agonist) and NSAIDs. The diarrhoea abated, but was followed by intense, unremitting right-sided colicky pain around the 75 km mark. The pain was constant for the remainder of the race but became unbearable upon finishing, prompting her presentation to the emergency centre.

Examination revealed a soft but distended abdomen with marked right-sided tenderness and voluntary guarding, without peritonism. The patient localised the pain to the right flank, radiating to the right back. The initial working diagnosis was renal colic or severe abdominal muscle spasm. Blood investigations and analgesia were ordered. Multimodal analgesia was commenced. Within the first hour, the patient received 1 g IV paracetamol, 3 mL of 99.9%/mL methoxyflurane inhaled, and 6mg IVI Morphine. Initial laboratory testing showed a neutrophil leucocytosis (white cell count 13.6 × 109/l), a mildly raised C-reactive protein, a lactate of 1.8 mmol/l and a high-sensitivity troponin I of 40.5 ng/l (reference < 21 ng/l), with a creatine kinase of 462 U/l. Renal function and electrolytes were near-normal, with a mildly raised urea consistent with exertion and dehydration. A point-of-care troponin elevation prompted a 12-lead electrocardiogram, which showed sinus rhythm with non-specific T-wave changes, and a bedside echocardiogram, which demonstrated good left ventricular function with no regional wall motion abnormality or structural lesion. An inadequate response to the above analgesia was followed by an analgesic dose of 30mg IVI Ketamine and prompted the ordering of a contrasted computed tomography (CT) of the abdomen.

CT demonstrated a markedly distended, gas-filled caecum measuring approximately 10 cm in diameter, displaced superomedially, with proximal small bowel dilatation and a decompressed distal colon - appearances consistent with caecal volvulus (Figure 1).^[4]^ There was no free intraperitoneal gas.

The patient was taken to theatre for emergency laparotomy. A midline laparotomy revealed an axially volvulated caecum that was significantly dilated but not ischaemic, with a dilated terminal ileum and a generally mobile colon with a redundant sigmoid. A right hemicolectomy with a stapled side-to-side ileocolic anastomosis was performed; the mesenteric defect was closed, and the abdomen was lavaged. Serial troponin testing after surgery showed a high-sensitivity troponin T within the normal range, and the biomarker rise was interpreted as exertional (type 2) demand ischaemia rather than an acute coronary syndrome.^[5]^

Postoperatively she was managed in the high-care unit and progressed well. Oral intake was reintroduced from the first postoperative day; she mobilised with physiotherapy, and the expected postoperative rise in C-reactive protein settled clinically. She was transferred to the general ward on the second postoperative day in a stable condition and made an uneventful recovery.

The patient was followed up via email 9 weeks post-presentation. She started walking back home upon arrival and began implementing a walk-run strategy as tolerated. She returned to running 7 weeks post-operatively and reports she is “listening to her body and running slower than she usually does”. She has entered and is training for a marathon due 30 weeks post-discharge and hopes to use it as a qualifier for the 2027 Comrades Marathon.

## Discussion

Caecal volvulus arises when a caecum with incomplete peritoneal fixation rotates around its mesenteric axis, producing a closed-loop obstruction.^[1]^ It accounts for a minority of colonic volvulus cases and characteristically affects women in the third to sixth decades, a demographic that fits the present patient. The contribution of endurance running is biologically plausible: repetitive mechanical jolting, increased intra-abdominal pressure and the marked alterations in splanchnic blood flow and bowel motility that accompany prolonged exertion may all promote torsion of a mobile caecum.^[3]^ Exercise-induced gastrointestinal syndrome, driven principally by splanchnic hypoperfusion, is well described and becomes clinically relevant beyond roughly two hours of sustained effort, a threshold far exceeded during a 90 km event.^[2]^ In this patient, self-medication with more than five loperamide tablets (a peripheral μ-opioid receptor agonist) and NSAIDs for early exercise-associated gastrointestinal symptoms during the race may have further slowed colonic transit and reduced mucosal perfusion. As to whether this may have been causative remains speculative.

Gastrointestinal complaints are common at the Comrades Marathon, but the overwhelming majority are benign and self-limiting; serious abdominal emergencies are rare and can be readily attributed to more common diagnoses, such as renal colic, as occurred initially here.^[2]^ The key learning point is that cross-sectional imaging should be used early and liberally in an endurance athlete with severe, localised abdominal pain that is disproportionate or persistent, because the CT signs of caecal volvulus - severe caecal distension and the whirl sign (Figure 2) - are both sensitive and decisive for management.^[4]^ A caecal diameter approaching or exceeding 10 cm signals a substantial risk of perforation and mandates urgent surgery.

Definitive management of caecal volvulus is operative. Resection with primary ileocolic anastomosis is preferred and is associated with the lowest recurrence, whereas detorsion or caecopexy alone carries higher recurrence rates and is generally reserved for selected circumstances.^[6]^ In this patient, early presentation and prompt imaging allowed resection before ischaemia supervened, contributing to an uncomplicated recovery. The transient troponin elevation is a recognised phenomenon after prolonged exertion and, in the presence of a structurally normal heart, should not delay necessary abdominal surgery.^[5]^

## Conclusion

Caecal volvulus is a rare but important cause of acute abdominal pain in an endurance athlete. A low threshold for CT imaging and timely surgical resection are essential to avoid caecal ischaemia and perforation, and clinicians caring for distance runners should keep this diagnosis in mind when right-sided abdominal pain is severe or persistent.

## Figures and Tables

**Fig. 1:**
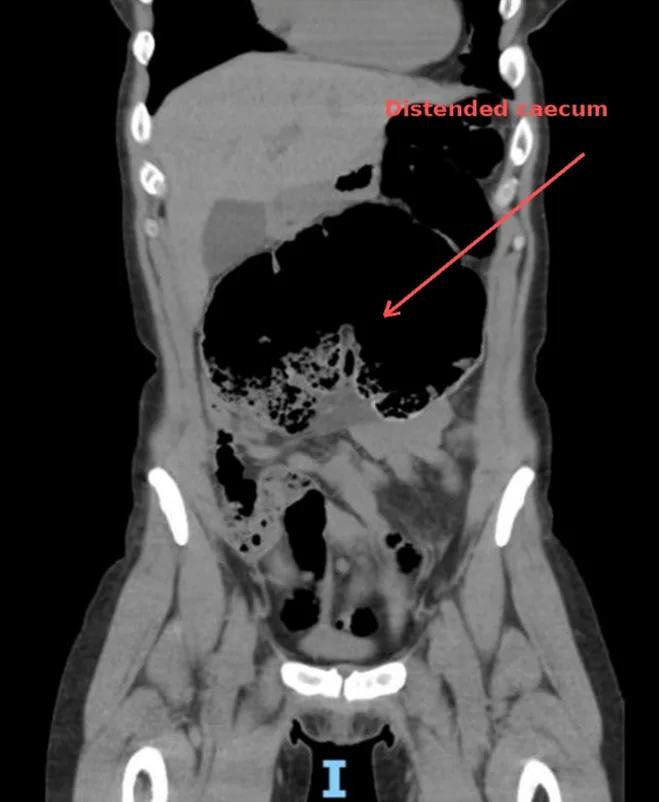
Coronal contrasted CT (computed tomography) of the abdomen showing a markedly distended, gas-filled caecum (arrow) displaced superomedially, with proximal small bowel dilatation and a decompressed distal colon, consistent with caecal volvulus.

**Fig. 2:**
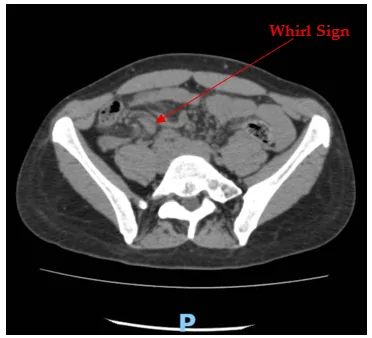
Axial contrasted CT (computed tomography) of the abdomen showing a “Whirl Sign” (arrow).
